# Nonlinear compositional and morphological evolution of ion irradiated GaSb prior to nanostructure formation

**DOI:** 10.1038/s41598-020-64971-9

**Published:** 2020-05-19

**Authors:** Michael A. Lively, Brandon Holybee, Michael Toriyama, Stefan Facsko, Jean Paul Allain

**Affiliations:** 1 University of Illinois at Urbana-Champaign, Urbana, IL 61801 US; 2 Intel Corporation, Hillsboro, OR 97124 US; 3 Northwestern University, Evanston, IL 60208 US; 4 Helmholtz-Zentrum Dresden-Rossendorf, Dresden, Germany; 5 Pennsylvania State University, State College, Pennsylvania, PA 16801 US

**Keywords:** Quantum dots, Characterization and analytical techniques, Surface patterning, Atomistic models

## Abstract

Low-energy ion irradiation of III-V semiconductor surfaces can lead to the formation of regular hexagonal dot patterns at the surface. We present experimental and computational results for ion irradiation of GaSb surfaces which elucidate the nature of the coupled compositional and morphological pattern-formation mechanisms. We demonstrate by *in-situ* grazing-incidence small-angle x-ray scattering (GISAXS) and angle-resolved Auger electron spectroscopy (ARAES) that the emergence of an altered compositional depth profile is essential to induce morphological changes at the surface. This morphological evolution of the surface follows nucleation-and-growth kinetics. Furthermore, we show from massive-scale molecular dynamics (MD) simulations that the compositional depth profile evolution leads to thermodynamic phase separation, providing a lateral compositional instability that drives pattern formation. Additionally, high-fluence simulations elucidate the irradiation-induced mechanisms of compositional depth profile formation. Prompt ion effects drive formation of single-element “protoclusters”, predominantly of Sb. Structural and energetic characterization of the simulation results indicate that Sb may be more mobile than Ga, providing a diffusional pathway for long-temporal-scale compositional evolution of the irradiated surface. Our findings motivate the development of new, comprehensive models which consider the total spatial and temporal complexity of multicomponent systems evolving under ion irradiation.

## Introduction

Since the discovery of regular and ordered nanoscale dot pattern formation induced on GaSb by Ar^+^ irradiation^1^, ion-induced pattern formation at the surfaces of III-V semiconductors has garnered significant experimental and theoretical attention. Since the initial observations, dot formation has also been seen on various other III-V surfaces^2,3^, and substantial work has focused on the influence of experimental parameters on the pattern characteristics^4,5^. *In-situ* characterization of irradiated III-V surfaces has demonstrated that a surface layer of altered composition emerges, and that compositional and morphological evolution are coupled to each other^6–10^. However, theoretical investigations of this coupled evolution have been divided in their conclusions. Several different models have been developed, considering mechanisms ranging from curvature-dependent sputtering which triggers preferential redistribution of one component along the surface^11,12^, to ion-induced compositional phase separation, where preferential sputtering from one phase results in a morphological instability^13,14^. Thus far, determining the dominant pattern-forming mechanism has proven difficult, as demonstrated by the fact that the models of Bradley^12^ and Norris^14^ predict identical morphologies but opposite lateral compositional gradients. More recent experiments have shown that the surface instability is “likely driven by chemical instability based on phase separation”^3^, but have not demonstrated a concrete mechanism which drives pattern formation.

Thus, a significant need exists for comprehensive atomistic simulations to study the nature of ion irradiation-induced compositional and structural mechanisms which drive nanopatterning. A key limitation of continuum models is a reliance on assumptions about which mechanisms drive the surface evolution. Atomistic computational models are not limited to a particular set of assumptions as continuum models are and are therefore able to consider all possible mechanisms without bias. These simulations can provide critical information about the prompt ion effects in ion-irradiated III-V materials, including preferential sputtering^15^, surface mass redistribution^16^, ion-induced defect production^17^ and accumulation^18^, and structural transformations^19^. Many of these mechanisms, notably the defect dynamics and structural transformations, have not been considered by any existing models of III-V nanopatterning, and their potential influence on the emergence of an altered compositional layer at the surface remains unclear at best. Additionally, the influence of the compositional depth profile on the prompt ion effects has not been characterized. These effects are critical to understand since they form the basis for mechanisms such as surface sputtering or irradiation-enhanced diffusion that drive surface transformation.

Here we present results from both experiments and simulations conducted to decipher the mechanisms which drive coupled compositional and morphological evolution of ion-irradiated III-V semiconductor surfaces. Experimentally, we have carried out *in-situ* characterization of the early stage evolution (Φ ≤ 3.4 × 10^16^ ions/cm^2^) Kr^+^ ion-irradiated GaSb surfaces by combining grazing-incidence small-angle x-ray scattering (GISAXS) and angle-resolved Auger electron spectroscopy (ARAES). Computationally, we have carried out two sets of massive-scale molecular dynamics (MD) simulations on the Blue Waters supercomputer^20^. The first set of simulations consisted of 500 eV Kr^+^ bombardment of a GaSb target with modified surface composition to a low fluence (Φ = 8.4 × 10^13^ cm^−2^) to investigate the effects of the compositional depth profile on the surface response to ion irradiation. The second set of simulations considered 500 eV Kr^+^ irradiation of initially pristine 50/50 GaSb to an experimentally relevant fluence (Φ = 7.5 × 10^15^ cm^−2^). These experimental and computational approaches elucidate the connection between ion-induced compositional depth profile disruption and the resulting long-scale lateral instability leading to pattern formation, which no approach thus far has been capable of demonstrating.

## Results

### *In-situ* characterization of morphological and compositional surface evolution

**Figure 1:**
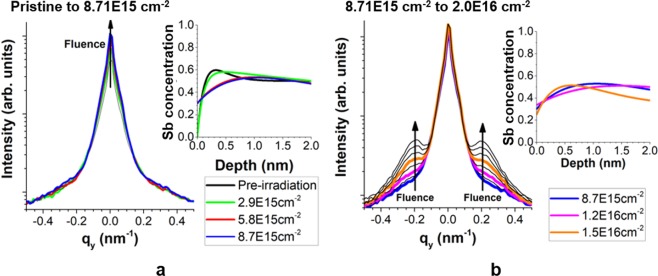
In-operando surface characterization from 500 eV Kr^+^ irradiation of GaSb. Large plots: in-operando GISAXS 1-D Yoneda wing plots for fluences (**a**) from zero to 8.7 × 10^15^ cm^−2^ and (**b**) from 8.7 × 10^15^ cm^−2^ to 2.0 × 10^16^ cm^−2^ (right). Insets: *in-situ* ARAES compositional depth profiles at indicated fluences.

We conducted experiments to characterize the early stages of ion beam irradiation-induced nanopatterning by 500 eV Kr^+^ ions incident on GaSb surfaces. We used *in-situ* grazing-incidence small-angle x-ray scattering (GISAXS) to investigate the topographical evolution and *in-situ* angle-resolved Auger electron spectroscopy (ARAES) to investigate the surface compositional evolution. The results are shown in Fig. 1 and indicate that the early-stage surface morphology evolution proceeds in two stages. In the first stage (Fig. 1(a)), a static state is observed in the GISAXS spectra from the start of irradiation up to a fluence of 8.7 × 10^15^ cm^−2^ with a slight increase in the ${q}_{y}=0$ peak height. This behavior is characteristic of irradiation-induced surface smoothening. In the second stage (Fig. 1(b)), shoulders appear in the GISAXS spectra at ${q}_{y}=\pm \,0.16$ nm^−1^, which correspond to scattering from periodic surface structures with spacing $\lambda =2\pi {q}_{y}^{-1}=39$ nm. These shoulders grow continuously in amplitude from a fluence of 8.7 × 10^15^ cm^−2^ up to the experimental ending fluence of 3.4 × 10^16^ cm^−2^. This behaviour is characteristic of surface nanostructure growth, and the two-part early-stage surface morphology evolution agrees well with previous experimental work^21^.

**Figure 2:**
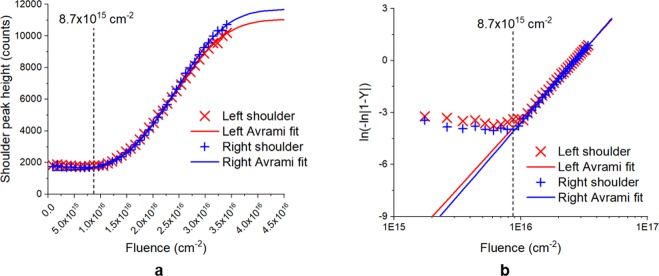
Result of fitting the GISAXS shoulder peak height data from Fig. 1 to the Avrami equation. (**a**) Comparison of fitted Avrami equation to experimental peak height data. (**b**) Linearized version of the same data presented to show the goodness of fit in the nanostructure growth fluence regime.

It is not clear from Fig. 1 whether the critical fluence of 8.7 × 10^15^ cm^−2^ corresponds to a *threshold fluence* at which the nanopatterning mechanism becomes active, or if it corresponds to a *crossover fluence* at which the dominant mechanism switches from smoothening to patterning. To clarify this, we fit the height of the GISAXS shoulder peaks (at ${q}_{y}=\pm \,0.16$ nm^−1^) to the Avrami equation, given by:

1 $$Y=\frac{h(\Phi )-{h}_{min}}{{h}_{max}-{h}_{min}}=1-\exp [-{(K\Phi )}^{n}]$$

where $Y$ is the transformation progress, $h$ is the shoulder peak height with ${h}_{min,max}$ being *fitted* minimum and maximum peak heights, Φ is the ion beam fluence, and $K$ and $N$ are fitted constants. The Avrami equation describes a phase transformation governed by nucleation-and-growth phase kinetics^22^. Therefore, we fit Eq. (1) to the GISAXS shoulder peak heights from Fig. 1(b) only, since the dominant surface morphology evolution in this fluence regime is nanostructure growth. The result of this fit is shown in Fig. 2. In addition to Eq. (1), we also show the linearized form of the Avrami equation:

2 $$\mathrm{ln}[-\,\mathrm{ln}(1-Y)]=n(\mathrm{ln}\,K+\,\mathrm{ln}\,\Phi )$$

which makes it clear that the fit is good in the growth regime ($\Phi  > 8.7\times {10}^{15}$ cm^−2^) but does not fit in the earlier smoothening regime ($\Phi  < 8.7\times {10}^{15}$ cm^−2^). We also note that attempts to fit Eq. (1) to the entire set of shoulder peak height data led to an overall poor fit. This is exactly as expected since surface smoothening kinetics are not described by the Avrami equation.

The goodness of the Avrami fit in the nanopattern growth regime indicates that the irradiation-induced nanopatterning is governed by nucleation and growth kinetics. In the earliest fluence regime, these mechanisms are in competition with and are dominated by irradiation-induced surface smoothening. The critical fluence of $\Phi =8.7\times {10}^{15}$ cm^−2^ is therefore a *crossover fluence* beyond which the nanostructure growth kinetics increasingly dominate over the surface smoothening kinetics. Growth kinetics continue to dominate over the remaining experimental fluence regime. Based on the Avrami fit we predict a saturation fluence (determined by $Y({\Phi }_{sat}) > 0.99$) of ${\Phi }_{sat}=4.2\times {10}^{16}$ cm^−2^, beyond which the surface nanostructures would cease to grow any further.

In addition to the GISAXS study, we performed *in-situ* ARAES to characterize the surface compositional depth profile evolution with fluence. These results are shown in the insets of Fig. 1, and details of the fitting and analysis of this data are given in the Supplementary Information. We observe a sharp transition in the compositional depth profile between $\Phi =2.9\times {10}^{15}$ cm^−2^ and $\Phi =5.8\times {10}^{15}$ cm^−2^, leading to an increase in Sb enrichment at the surface and a corresponding decrease in Sb concentration in the sub-surface. For fluences $\Phi \ge 8.7\times {10}^{15}$ cm^−2^, corresponding to the growth-dominant fluence regime of the surface morphology evolution, we observe a fairly stable compositional depth profile up to a fluence of $\Phi \approx 1.5\times {10}^{16}$ cm^−2^, beyond which ARAES is not able to accurately characterize the surface compositional depth profile due to the increase in surface roughness. Taken together, these observations indicate that the surface compositional depth profile evolution serves as a precursor to nanostructure growth at the surface. If the nanostructure growth is characterized by nucleation and growth kinetics, it follows that the compositional depth profile evolution is closely tied to the nanopattern nucleation kinetics. In the following section, we present the results of atomistic simulations to elucidate this connection.

### Simulated ion irradiation of altered composition GaSb

**Figure 3:**
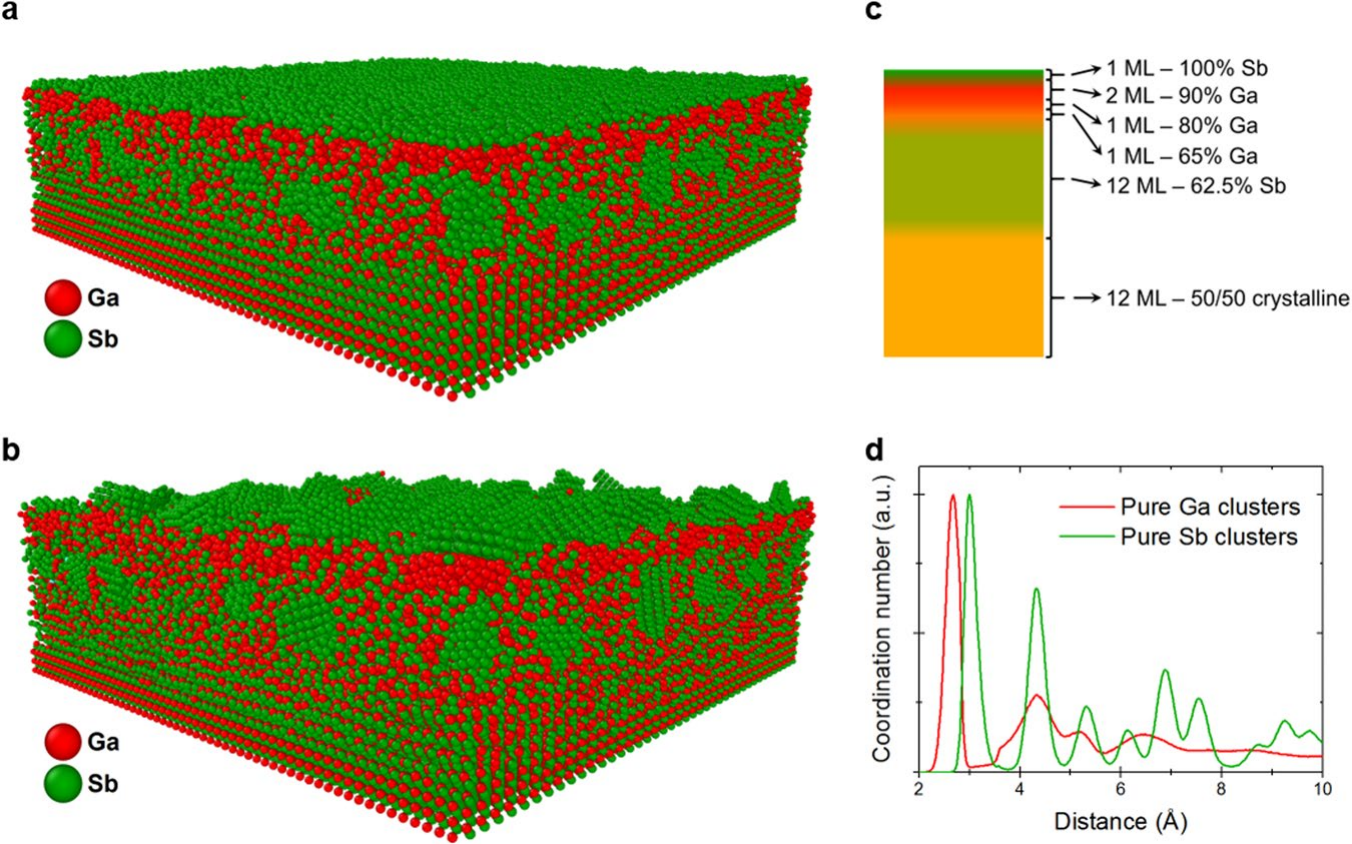
Results from MD simulations of 500 eV Kr^+^ ion irradiation of the GaSb surface with a prescribed compositional depth profile. (**a**) Initial snapshot of the prepared surface. Only a 25×25 nm^2^ section of the complete 100 × 100 nm^2^ surface is shown. (**b**) Snapshot showing the final state of the same surface after bombardment to a fluence of 8.4 × 10^13^ Kr^+^ ions/cm^2^. (**c**) Schematic of the prescribed compositional depth profile for the simulated surfaces. Layer thickness is given in monolayers (ML), with 1 ML = 0.22 nm. (**d**) Normalized radial coordination analysis of pure-Ga and pure-Sb clusters in the surface shown in (**b**), indicating crystalline Sb clusters and amorphous Ga clusters. Snapshots (**a**,**b**) were created with the OVITO software tool^23^.

We performed molecular dynamics (MD) simulations of 500 eV Kr^+^ irradiation on a GaSb surface with a modified compositional depth profile. The results of these simulations are shown in Fig. 3. The explicit purpose of these simulations is to model the fully three-dimensional compositional dynamics arising from the compositional depth profile elucidated by experiments. In other words, these simulations are used to extend the experimental results by connecting the finding of a compositional *depth* profile to a *lateral* compositional gradient which can drive the pattern-forming surface instability.

These simulations covered a surface size of 100 × 100 nm^2^ (2.4 million atoms) and reached a cumulative ion fluence of 8.4 × 10^13^ cm^−2^ (8,400 cumulative impacts). The computational scale of this study was much greater than that of any previous MD study of ion-surface interaction, and therefore these simulations required the use of the Blue Waters supercomputer at the University of Illinois. This extreme scale was chosen to capture potential ion-irradiation-driven mechanisms on the lateral scale of experimentally observed spacing (50–80 nm) of nanodots formed on GaSb by ion irradiation. The details of the compositional depth profile are shown in Fig. 3(c).

The most apparent effect of the prepared compositional depth profile is phase separation leading to cluster formation in layers of altered stoichiometry. This phase separation occurs even before the irradiation simulations, during the surface preparation, as shown in the pre-irradiation surface snapshot of Fig. 3(a). This is most evident in the 62.5% Sb-enriched surface sub-layer, which separates into Sb nanoclusters 1–2 nanometres in diameter surrounded by stoichiometric GaSb. Similar formation of Ga nanoclusters is observed for the Ga-enriched layer, although this is not easily visible due to the small thickness (0.86 nm) of that layer.

During the simulations, we observed rapid self-organization of the Sb nanoclusters (and surface monolayer) into crystalline lattices over a sub-nanosecond timescale. This rapid Sb cluster crystallization is clearly visible in Fig. 3(b). Radial distribution function (RDF) analysis of atoms in Ga and Sb clusters, shown in Fig. 3(d), confirms the simple-cubic ordering of the Sb clusters as evidenced by the well-defined peak pattern of the Sb cluster RDF. In contrast, the relative lack of peaks in the Ga cluster RDF indicate that the Ga clusters remain amorphous. The observation of crystalline Sb clusters is not unprecedented, as previous experimental studies of ion-irradiated GaSb nanowires have found evidence of crystalline Sb cores surrounded by GaSb shells^24^. We also note that for Sb, the simple cubic lattice has a number density of 3.4 × 10^22^ cm^−3^ compared to 3.2 × 10^22^ cm^−3^ for the rhombohedral lattice. The simple cubic structure is therefore favoured over the rhombohedral structure for the Sb nanoclusters to minimize compressive stress in the GaSb surface.

It is necessary to clarify whether the rapid crystallization of the Sb nanoclusters is an irradiation-induced mechanism or an intrinsic material response of Sb. To address this question, we also conducted a simulation of a GaSb surface with the same compositional depth profile but simply allowed the surface to evolve for ~4 nanoseconds without any ion irradiation. From this simulation we observed the exact same transformation of the Sb clusters as shown in Fig. 3, indicating that the crystallization of Sb is not directly driven by the ion irradiation. This is expected, since Sb recrystallizes efficiently even for temperatures below the simulated temperature of 300 K^25^. Note that the Sb crystallization is still *indirectly* an irradiation-driven phenomenon, since it occurs due to the irradiation-induced compositional depth profile which drives the phase separation and Sb nanocluster formation.

The significance of these simulations is that they extend the experimental results by connecting the experimentally observed compositional depth profiles to a *lateral* compositional and structural inhomogeneity via phase separation and rapid crystallization of Sb nanoclusters. This lateral compositional and structural inhomogeneity is a surface response to the irradiation-induced compositional depth profile and is necessary to drive morphological instability at the surface. Recalling our experimental analysis of the surface morphology evolution via GISAXS and the Avrami equation, the Sb nanoclusters can serve as nucleation “seeds” which drive the growth of surface nanostructures as ion irradiation proceeds to higher fluences. Thus, the observation of Sb phase separation and nanocluster formation fits well with the nucleation and growth kinetics inferred from GISAXS measurements.

Here we must make two clarifications. First, we note that the compositional depth profile used in these simulations does not exactly match those measured in experiments by ARAES. The reason for this is that the depth profiles for the simulations were constructed based on an unrefined, preliminary analysis of the ARAES data. However, the simulation results remain valid and compelling because phase separation and nanocluster formation are observed in any Ga-enriched or Sb-enriched layer, regardless of the composition of neighbouring layers. This is demonstrated in Supplementary Fig. S6 and in previous work by Albe and coworkers^26^. Secondly, we also note that the ion fluence used in these simulations was too small to observe any significant irradiation-induced or irradiation-driven mechanisms (for comparison, the fluence between GISAXS scans in our experiments was 8.4 × 10^14^ cm^−2^, an order of magnitude greater than our simulated fluence). Therefore, the question of which *directly* irradiation-driven mechanisms contribute to the surface compositional and morphological evolution remains an open one. We address this knowledge gap below.

### Deciphering prompt ion effects in pristine GaSb

We conducted a second set of MD simulations for cumulative ion irradiation with 500 eV Kr^+^ to a fluence of 7.5 × 10^15^ cm^−2^ on initially pristine GaSb(110) surfaces, with the surface size reduced to 25 × 25 nm^2^. We note explicitly that this fluence, in contrast to the fluence reached in the previous set of simulations presented above, is of similar order to the crossover fluence identified from the experimental results (see Fig. 2). Therefore, the results from these simulations are considered representative of the *irradiation-induced* effects on the GaSb surface under experimental conditions. For these simulations, the intention was to elucidate the prompt ion-induced mechanisms in the surface which can drive formation of the compositional depth profile observed in experiments (see Fig. 1).

**Figure 4:**
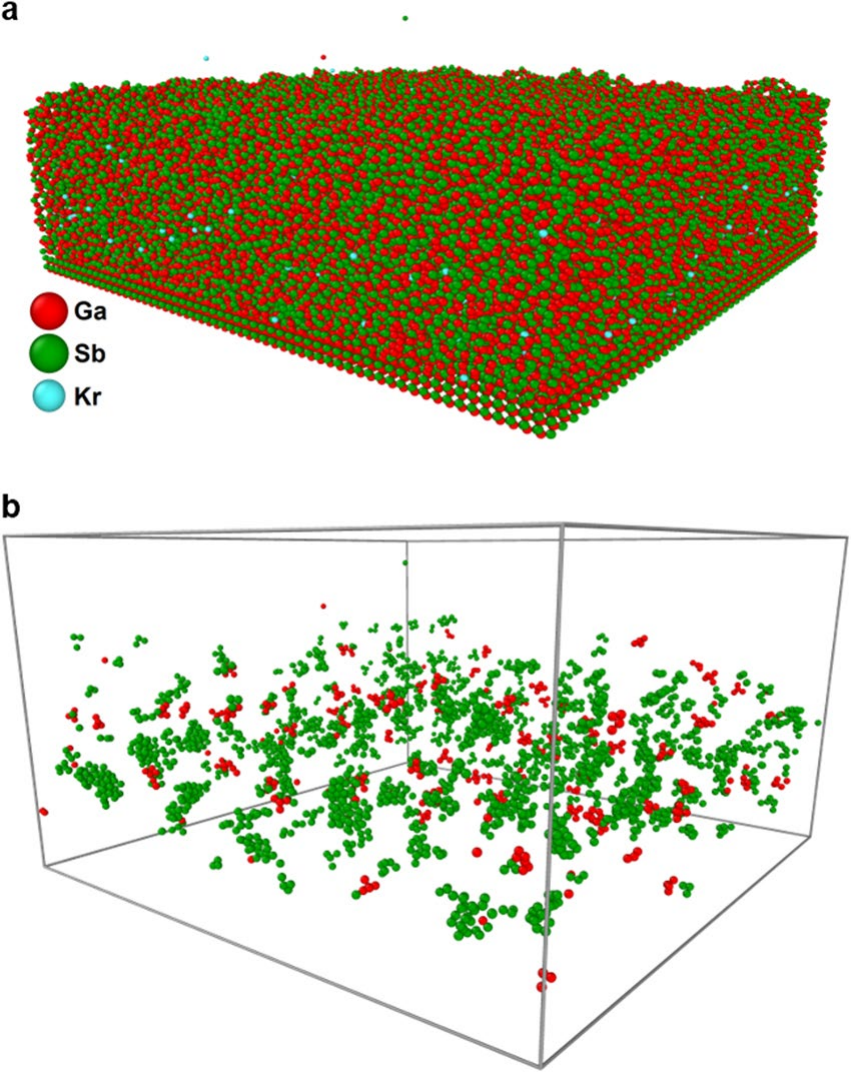
Results from MD simulations of 500 eV Kr^+^ ion irradiation of the initially pristine GaSb surface. (**a**) Snapshot of the simulated surface after bombardment with 500 eV Kr^+^ to a fluence of 7.5×10^15^ ions/cm^2^. (**b**) Snapshots of the same surface showing only Ga clusters (red) and Sb clusters (green). Snapshots were created with the OVITO software tool^23^.

Snapshots of the simulation results are shown in Fig. 4. Strikingly, we observe formation of both Sb and Ga clusters during the ion irradiation, as seen in Fig. 4(b). Two kinds of clusters exist in the surface: the first kind of clusters are those consisting of only a few (~4 to 8) atoms, which are most likely substitutional complexes and are formed by both Ga and Sb; the second kind of clusters, which are formed only by Sb atoms, are large formations containing dozens of atoms with typical diameters of about 1 nanometre which we refer to as “protoclusters” to differentiate from the clusters seen in Fig. 3. Since only Sb atoms form protoclusters, the number of Sb atoms in clusters is 5 to 10 times greater than the number of Ga atoms in clusters at any given fluence.

**Figure 5:**
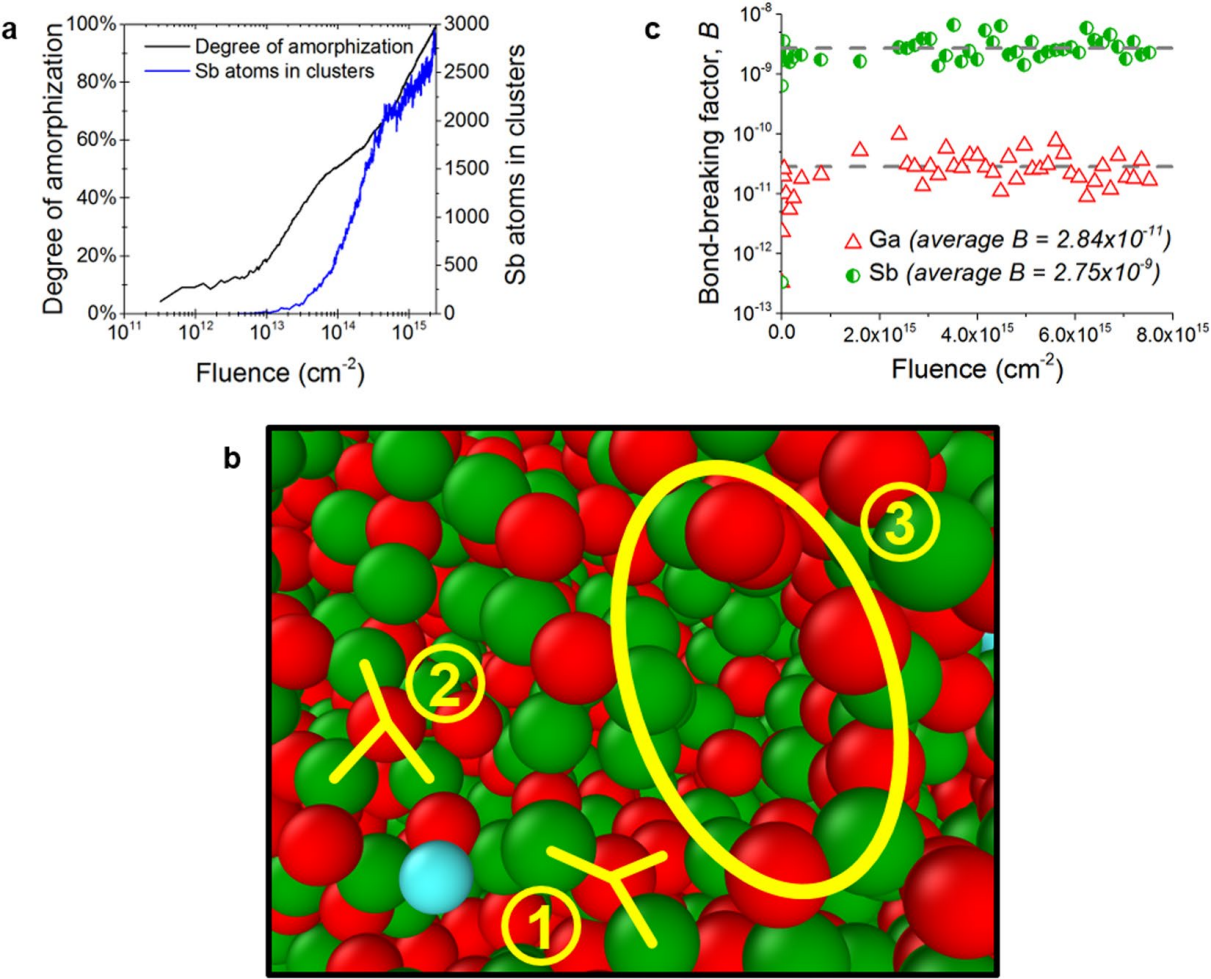
Analysis of MD simulation results for 500 eV Kr^+^ irradiation of GaSb. (**a**) Surface amorphization progress and number of Sb atoms in protoclusters with respect to ion fluence. (**b**) Close-up cutaway snapshot (red = Ga, green = Sb, blue = Kr) of the ion-bombarded surface after a fluence of 6.1 × 10^15^ cm^−2^, with several features indicated: (1) “flat” and (2) “angled” three-neighbour bonding states with line segments indicating each bond; (3) a small void within the surface. (**c**) Bond-breaking factors for Ga and Sb calculated for increasing values of the ion fluence. Snapshot (**b**) was created with the OVITO software tool^23^.

The Sb protoclusters are formed solely by the *prompt ion effects* during irradiation, i.e. they are formed out of the damage and disorder caused by successive ion-induced collision cascades in the surface. We demonstrate this in Fig. 5(a) by characterizing the surface disorder in terms of the irradiation-induced surface amorphization and comparing this against the number of Sb atoms in protoclusters for ion fluences up to 2.4 × 10^15^ cm^−2^. We compute the amorphization progress (*Am%*) of the surface by comparing the radial distribution function (RDF), denoted as $g(r)$, at a given fluence to the RDFs of the initial (Φ = 0) and final (Φ = 7.5 × 10^15^ cm^−2^) surface states, with the definition given as:

3 $${\Delta }^{2}=\sum _{RDF}{[g(r)-{g}_{init}(r)]}^{2}$$

4 $$Am \% =\sqrt{\frac{{\Delta }^{2}-{\Delta }_{final}^{2}}{{\Delta }_{final}^{2}}}$$

The data in Fig. 5(a) shows that the ion-irradiated surface quickly becomes highly disordered, surpassing 50% amorphization for fluences as low as 2 × 10^13^ cm^−2^. By contrast, a significant degree of protocluster formation does not occur until the fluence approaches 10^14^ cm^−2^, after the surface has already become more than 60% amorphous. This indicates that *structural* disorder induced by the prompt ion effects is a prerequisite for the emergence of *chemical* or compositional disorder, i.e. protocluster formation.

The nature of the ion-induced surface disorder is visualized in Fig. 5(b). Interestingly, most atoms in the surface (both of Ga and Sb) have only three nearest neighbours which they are bonded to. This contrasts with what would be expected from a typical amorphous GaSb surface, in which the tetrahedral-amorphous structure (ta-GaSb) would be expected. This therefore indicates a radical, irradiation-induced structural shift within the GaSb surface. As shown in Fig. 5(b), the three-neighbour atoms exist in two broad classifications. In the “flat” configuration (label ‘1’ in Fig. 5(b)), the central atom is directly centred between the three atoms and the configuration forms a trigonal geometry. In the “angled” configuration (label ‘2’ in Fig. 5(b)), however, the central atom is offset from its bonded neighbours, forming a tetrahedral configuration with one bond missing or dangling. In crystalline point-defect terms, the dangling bond in this latter configuration is a vacancy site. In addition, small voids or pores are also formed over the course of the ion bombardment (label ‘3’ in Fig. 5(b)), which are typical for an amorphous structure.

As a first-order approximation to quantify the effects of the ion-induced surface disorder, we calculate a so-called “bond-breaking transition factor” (BBTF) based on the per-bond potential energy of the surface atoms. This factor represents the probability for an atom of a given type to undergo a bond-breaking transition and can be used as a first estimate of the per-species atomic mobility or diffusivity. From the simulation data, the energy per bond ${E}_{b}$ is extracted and used to compute the bond-breaking factor for each element in the surface, defined by

5 $${B}_{Ga,Sb}=\frac{1}{{N}_{Ga,Sb}}\sum _{Ga,Sb}{b}_{i}=\frac{1}{{N}_{Ga,Sb}}\sum _{Ga,Sb}{\rm{\exp }}\left(\frac{{E}_{b,i}}{{k}_{B}T}\right)$$

where $T$ is the simulation temperature (300 K) and $N$ is the total number of atoms of the subscripted type. The BBTF are calculated for Ga and Sb and shown in Fig. 5(c). The fluence-averaged BBTF for Sb is two orders of magnitude higher than that of Ga, indicating that Sb is far more likely to undergo bond-breaking transitions. These transitions may be prompt collisional effects leading to increased defect formation during ion impacts, particularly of Sb vacancies which then mediate fast Sb diffusion. Alternately, these transitions may drive the surface kinetics on the long temporal scale between ion impacts by lowering energy barriers for Sb atomic motion. At either timescale, the elevated bond-breaking transition rate for Sb atoms therefore implies a higher Sb atomic mobility relative to that of Ga.

The BBTF concept enables us to connect the prompt ion effects observed from the MD simulations to the long temporal scale which cannot be directly accessed by MD. This connection is critical because even though the *fluence* (7.5 × 10^15^ cm^−2^) is on the same order as the experimental crossover fluence, the physical *timescale* of the simulations is on the order of microseconds, much smaller than the experimental timescale of seconds to minutes. Thus, the fact that a compositional depth profile resembling experimental measurements did not form during the simulations (Fig. 4(a)) means that at least one key mechanism driving the compositional instability is only active on a long temporal scale beyond our MD simulations. The high Sb mobility indicated in Fig. 5(c) implies a strong asymmetric diffusivity of Sb through the irradiated surface relative to Ga. Referring to the earlier discussion of nucleation and growth kinetics, if prompt, collisional ion effects serve as the nucleation mechanism for Sb protoclusters, then enhanced Sb diffusivity provides a growth mechanism for the protoclusters *via* fast transport of Sb atoms to the protocluster/GaSb interface. We note that in fact, the Sb protoclusters are not stable in our simulations but are continually broken and re-formed as irradiation continues. Thus, this enhanced Sb diffusion mechanism is necessary to grow the Sb protoclusters to stable sizes (i.e. few-nanometre diameter, as in Fig. 3) at which point they can contribute to the surface morphological instability leading to nanostructure formation.

To conclude our presentation of the simulation results, we must address two further points. First, we note that previous experimental^27^ and computational^28^ studies of diffusion in GaSb have indicated that Ga has greater mobility than Sb in (crystalline) GaSb by several orders of magnitude. However, these studies have been restricted to near-equilibrium conditions, whereas the surface under ion irradiation is driven very far from equilibrium in our simulations. The highly disordered ion-irradiated surface is a vastly different environment in terms of concentration and distribution of defects which mediate the atomic diffusion. Thus, we should expect diffusion rates in the ion-irradiated surface to differ markedly from their counterparts in equilibrium conditions. Secondly, we must note that the bond-breaking transition factor given here is only a first estimate of the relative mobilities of the two species. A proper calculation of the atomistic diffusivity of Ga and Sb requires a precise characterization of the ion-induced point defect distribution in the surface, since diffusion at the atomic scale is mediated by point defects. The nature of the irradiation-produced defect distribution is unclear and remains the subject of future investigations.

## Discussion

The complementary experimental and computational results presented here give a complex picture of the physical mechanisms underlying irradiation-driven III-V semiconductor surface modification and nanopatterning. Experimentally, we have shown a nonlinear temporal relationship between composition and morphology in the early stages of irradiation. Compositionally, the surface develops and evolves a compositional depth profile with varying surface and sub-surface component enrichment. Morphologically, nonlinear pattern formation and growth kinetics occur which follow an Avrami law. We have also carried out complementary simulations which both extend and explain the experimental results. On one hand, we have shown that the compositional depth profile leads to lateral phase separation in the enriched regions, providing a path to surface morphological instability. On the other hand, we have identified irradiation-induced Sb protocluster formation along with fast diffusional mobility of Sb through the sub-surface as probable mechanisms driving compositional evolution of the surface. This three-dimensional spatial and temporal complexity in the early stages of surface irradiation has been hitherto unexplored.

Taken as a whole, these findings elucidate the connection between prompt ion-surface interactions and gradual mechanisms which act at long temporal scales. At long temporal scales, the enhanced mobility of Sb provides a mechanism for the growth of the Sb protoclusters which are initially formed by prompt ion effects. The Sb protocluster nucleation and growth kinetics potentially contribute to the larger surface compositional and morphological evolution. Compositionally, Sb protoclusters act as sinks for mobile Sb atoms, removing Sb from the surrounding GaSb and thus further driving surface compositional evolution. Morphologically, stabilized Sb clusters will be exposed at the surface by continued ion sputtering. The sputtering yield difference between laterally-distributed Sb and GaSb surface phases can drive surface instability and nanopattern formation^13,14^. Therefore, the results from our MD simulations provide a physical basis to model the surface compositional and morphological evolution, and ultimately to develop a *predictive* model of III-V surface nanopatterning by ion irradiation.

In contrast, theoretical models of ion-induced surface nanopatterning at III-V surfaces, i.e. Bradley-Harper (BH) theory^29^ and its extensions^12–14^, are unable to predictively model this phenomenon. The major reason for this is that these models are only two-dimensional in space and linear in time, a scope which is insufficient to capture the significant results we present here. No existing model can capture the three-dimensional compositional dynamics of depth profile evolution nor of sub-surface cluster formation, nor do these models incorporate in any way the structural transformation and irradiation-induced defect distribution within the surface. Furthermore, while nonlinear extensions of BH theory have been developed for high fluences, no existing approach captures the compositional or morphological nonlinearity at low fluences which result from the Avrami-type kinetics our results have demonstrated. In addition to physical limitations, we also note that BH-type models rely on arbitrary tuned coefficients, which are difficult if not impossible to relate directly to experimentally relevant physical parameters. These models are thus unable to *predict* the surface morphology for a particular combination of ion species, target material, and experimental parameters. In light of these shortcomings, this work should strongly motivate development of new, predictive modelling paradigms which can incorporate the revealed spatial and temporal complexity and move beyond existing, inadequate modelling conventions.

The experimental and computational studies given here provide a physical basis for such a paradigm. In particular, the importance of ion-induced structural disorder in governing the irradiation-driven mobility of the component species should be a key component of next-step modelling following from this work. Along these lines, we anticipate the development of a highly versatile class of models based on these types of mechanisms, which can be generalized for irradiation-induced modification of any complex, multicomponent surfaces. One particularly relevant class of target materials appropriate to study with these models are ion-irradiated high-entropy alloys (HEAs)^30^ – promising materials with unique structural and mechanical properties for which the nature of irradiation-induced surface modification remains very much an open question.

## Methods

### Experimental methods

GISAXS experiments were performed at the National Synchrotron Light Source 1 (NSLS 1) at Brookhaven National Laboratory in the X-21 beamline. An UHV chamber equipped with a goniometer, photodetector, and a broad beam ion source was setup at the end of the beamline allowing monochromatic, 10 keV X-rays to interact with a loaded GaSb sample. The goniometer was used to offset the GaSb sample by 0.2° in order to perform the GISAXS characterization. A complete description of the GISAXS setup and geometry is given in Zhou *et al*.^31^. The GaSb sample was initially etched with HCl in order to remove the native oxide layer, cleaned with methanol, and immediately loaded into the UHV chamber to prevent oxide formation. *In-situ* GISAXS was performed every 15 seconds during the 500 eV Kr^+^ ion irradiation, resulting in incremental fluence steps of 8.4 × 14 cm^−2^ between GISAXS scans.

The ARAES measurements were done at the Ion Beam Center of the Helmholtz-Zentrum Dresden-Rossendorf in Dresden, Germany, using a Microlab 310 AES instrument by Thermo Fisher. A total of 9 different sample-to-analyser angles were used for each measurement: 0°, 15°, 30°, 45°, 67.5°, 75°, 82.5°, 85.5° and 87.5°. A mathematical model of the compositional depth profile was developed in order to fit the ARAES data, which is described in the Supplementary Information.

### Simulation procedure

Our MD simulations were carried out with the LAMMPS package^32^ on the Blue Waters supercomputer at Illinois^20^. The generalized simulation conditions are shown in more detail in Supplementary Fig. S3. For the first set of simulations, the simulation environment consists of a 100 × 100 × 6.25 nm^3^ GaSb surface containing 2,237,216 atoms. For the second set of simulations, the simulation environment consists of a 25 × 25 × 7.6 nm^3^ crystalline GaSb(110) surface containing 175,972 atoms, with additional layers of GaSb(110) added to the bottom of the surface at periodic intervals throughout the simulation. The target is then irradiated with 500 eV Kr^+^ ions which are generated in random locations >1 nm above the surface with a downward trajectory. Ga-Ga and Sb-Sb interactions are modelled with a modified version of the Tersoff-ZBL hybrid potential developed by Albe and coworkers^26^, while Ga-Sb interactions are treated with the Tersoff potential given by Powell *et al*.^33^. All interactions with ion species are modelled by the purely repulsive ZBL potential^34^. The boundary conditions are periodic in the lateral dimensions and fixed in the vertical dimension. The bottom 0.86 nm (4 ML) of the surface are held fixed, while the next-lowest 1.72 nm (8 ML) of the surface are held at a temperature of 300 K by a Berendsen thermostat^35^. Sputtered and reflected particles are removed from the top of the simulation volume.

### Constructing the surface with altered composition

The surface with an altered compositional profile (Fig. 3(a)) is constructed from bottom to top by creating and amorphizing one layer at a time. For each successive layer, atoms are generated and constrained by reflecting walls at the minimum and maximum z-coordinates for that layer. The atoms are heated to a temperature between 2500 and 3500 K, depending on the layer composition, and then are immediately thermalized at 300 K. This procedure leads each layer to an amorphous state similar to that resulting from ion irradiation. After all layers are created, the surface is relaxed at 300 K for an extended time to allow the whole surface to reach equilibrium. Due to the computational expense of this procedure, the surface is prepared as a 25 × 25 nm^2^ area which is then duplicated in a 4 × 4 array to obtain the final 100 × 100 nm^2^ surface.

Additional details of this procedure, including several illustrative figures, are given in the Supplementary Information.

### Cluster and coordination analysis

We use the Open Visualization Tool (OVITO)^23^ to identify atoms in clusters and carry out coordination analysis of the MD simulation data. Clusters are defined as any group of atoms of one type which meet one of two criteria: (1) they do not have a neighbour of another type, or (2) they are neighbours of one or more atoms which meet the first criteria.

## Supplementary information

Supplementary Information.

## Data Availability

The data generated and analysed during this study are available from the corresponding author on reasonable request.
