# Supplementary material for: Nonlinear compositional and morphological evolution of ion irradiated GaSb prior to nanostructure formation
Source: Sci Rep. 2020 May 19;10:8253. doi: 10.1038/s41598-020-64971-9 (PMC7237666; doi:10.1038/s41598-020-64971-9)
Supplement: Supplementary file 1 — Supplementary Information. [file 41598_2020_64971_MOESM1_ESM.pdf]

SUPPLEMENTARY INFORMATION

For the manuscript entitled

Nonlinear compositional and morphological evolution of ion irradiated GaSb prior to nanostructure formation

Michael A. Lively[1*] (ORCID: 0000-0001-6511-9852), Brandon Holybee[2], Michael Toriyama[3], Stefan Facsko[4] (ORCID: 0000-0003-3698-3793), and Jean Paul Allain[5]* (ORCID: 0000-0003-1348-262X)

[1]*University of Illinois at Urbana-Champaign, Urbana, IL 61801*

[2]*Intel Corporation, Hillsboro, OR 97124*

[3]*Northwestern University, Evanston, IL 60208*

[4]*Helmholtz-Zentrum Dresden-Rossendorf, Dresden, Germany*

[5]*Pennsylvania State University, State College, PA 16801*

Author information
M.A.L. mlively2@illinois.edu
B.H. bholybee@gmail.com
M.T. michaeltoriyama2024@u.northwestern.edu
S.F. s.facsko@hzdr.de
J.P.A. allain@psu.edu

GISAXS analysis. The experimental setup for GISAXS characterization has been described in the main text Methods section and elsewhere[1]. We briefly explain here the procedure to analyse the GISAXS data and extract the curves shown in **Figure 1** of the main text. The GISAXS raw data consists of scattered x-ray intensity measurements over the two-dimensional (q_y, q_z) domain. The *1-D Yoneda wing* is the intensity curve over q_y located at the value of q_z which corresponds to the highest spectral reflectivity of the sample surface. This is illustrated by an example in **Supplementary Figure S1** for clarity. The 1-D Yoneda wing is obtained at each fluence step at which a GISAXS scan of the surface was taken to obtain the fluence-dependent plots such as those in **Figure 1** of the main text. For 500 eV Kr^+ irradiation of GaSb, GISAXS scans were taken at fluence steps of 8.37×10^{14} cm^{-2} starting with the initial surface and proceeding up to an ending fluence of 3.41×10^{16} cm^{-2}.

ARAES analysis. We obtain compositional depth profiling data from ARAES using a novel phenomenological approach described here. To obtain angle-resolved measurements, we rotate the sample surface relative to the detector and conduct AES measurements at nine different angles: 0°, 15°, 45°, 60°, 67.5°, 75°, 82.5°, 85.5°, and 87.5°. This procedure is schematically shown in **Supplementary Figure S2**, which also shows how the surface rotation influences the *effective* mean free path, i.e. the average depth from which a detected Auger electron originates. This effective mean free path varies with detector angle as $\lambda_{eff} = \lambda_0 \cos\theta$. In other words, as the detector angle relative to the surface normal increases, the average depth from which a detected Auger electron is emitted decreases. This principle enables depth-resolved compositional measurements to be obtained.

We then develop a mathematical model for the compositional depth profile evolution. This model is based on work by Sigmund and Lam[2] which describes the compositional depth profile evolution in terms of surface recession (i.e. preferential sputtering) and non-preferential diffusion current. We incorporate additional fluence dependence of the model coefficients giving rise to an expression:

$$\frac{\partial c_i(z,\Phi)}{d\Phi} = w(\Phi)\frac{\partial c_i}{\partial z} + D(\Phi)\frac{\partial^2 c_i}{\partial z^2} \quad \text{(S1)}$$

Here we have: c_i is the compositional depth profile for species i which depends on depth z and fluence Φ, $w(\Phi)$ is the surface recession coefficient, and $D(\Phi)$ is the diffusion coefficient. This equation is subject to the boundary condition determined by the partial sputtering yield Y_i of species i:

$$w(\Phi)c_i(z=0,\Phi) + D(\Phi)\frac{\partial c_i}{\partial z}\bigg|_{z=0} = Y_i(\Phi) \quad \text{(S2)}$$

We now introduce our phenomenological approach to solve these equations. First, we assume that the coefficients $w(\Phi)$, $D(\Phi)$, and $Y_i(\Phi)$ vary slowly with fluence. Thus, while they have different values at each ARAES measurement fluence, they can be treated as approximately constant with respect to fluence. We therefore index these by fluence step j i.e. as w_j, D_j, and $Y_{i,j}$

respectively. Secondly, we introduce a functional form for the slowly varying derivative with respect to fluence which we take to be a decaying exponential:

$$\left.\frac{\partial c_i}{\partial \Phi}\right|_{\Phi=\Phi_j} = f_j(z) = Ae^{-\gamma z} \quad \text{(S3)}$$

We now have an ordinary differential equation which can be solved at each fluence step:

$$Ae^{-\gamma z} = w_j \frac{\partial c_{i,j}}{\partial z} + D_j \frac{\partial^2 c_{i,j}}{\partial z^2} \quad \text{(S4)}$$

This is easily solved to obtain an analytical form of the compositional depth profile with five fitted parameters:

$$c_{i,j}(z) = k_1 e^{-\beta_1 z} + k_2 e^{-\beta_2 z} + k_3 \quad \text{(S5)}$$

We note that this phenomenological approach is only valid to a depth of $z \leq 2$ nm, as beyond this depth collisional mixing and damage from the implanted ions becomes significant which is not included in our model. Since we are most interested in the near-surface composition, this is an acceptable limitation.

Finally, we fit Supplementary Equation S5 to the ARAES data for each fluence. We first eliminate background counts from the raw data using the Savitsky-Golay method[3]. This leaves us with a *convoluted* compositional measurement which is the detected Auger electron intensity $I_{i,j}(\theta)$. This measurement is convoluted because the measured intensity includes Auger electrons distributed over various origination depths. The convoluted intensity value is related to the actual depth profile by:

$$I_{i,j}(\theta) = \frac{A_0 \sigma_{DF} \sigma_{XF}}{\cos\theta} \int_0^\infty c_{i,j}(z) e^{-\frac{z}{\lambda_0 \cos\theta}} dz \quad \text{(S6)}$$

where the prefactor includes a constant A_0, detector efficiency factor σ_{DF}, and excitation factor σ_{XF}. Noting that we cannot assume that the initial cleaved-*in-vacuo* surface has a uniform 50/50 composition, we use a *relative* fitting procedure by comparing the ratio between Ga and Sb Auger electron intensities at each detector angle:

$$\frac{1}{\sigma_{SF}} \frac{\lambda_{Sb,0} I_{Sb,j}(\theta)}{\lambda_{Ga,0} I_{Ga,j}(\theta)} = \frac{\int_0^\infty c_{Sb,j}(z) e^{-\frac{z}{\lambda_{Sb,0}\cos\theta}} dz}{\int_0^\infty [1 - c_{Sb,j}(z)] e^{-\frac{z}{\lambda_{Ga,0}\cos\theta}} dz} \quad \text{(S7a)}$$

$$\sigma_{SF} = \frac{I_{Sb,0}(\theta)}{I_{Ga,0}(\theta)} \quad \text{(S7b)}$$

Note that the sensitivity factor implicitly incorporates the differences in detector and excitation factors between Ga and Sb. The final form of the fitting function used to obtain the coefficients of Supplementary Equation S5 is then

$$\frac{1}{\sigma_{SF}}\frac{\lambda_{Sb,0}I_{Sb,j}(\theta)}{\lambda_{Ga,0}I_{Ga,j}(\theta)} = \frac{-\frac{k_1}{\beta_1 + \Gamma_{Sb}(\theta)} - \frac{k_2}{\beta_2 + \Gamma_{Sb}(\theta)} + k_3\Gamma_{Sb}(\theta)}{1 + \frac{k_1}{\beta_1 + \Gamma_{Ga}(\theta)} + \frac{k_2}{\beta_2 + \Gamma_{Ga}(\theta)} - k_3\Gamma_{Ga}(\theta)} \quad \text{(S8)}$$

where we have used $\Gamma_i(\theta) = \left[\lambda_{i,0}\cos\theta\right]^{-1}$ for brevity. The relative fitting procedure is necessary to account for the fact that the initial surface does not have a uniform 50/50 depth profile. However, this approach does reduce the number of data points available for fitting by half, from eighteen (nine angles and two elements) to nine (the ratio of Ga and Sb intensities at nine angles), which does introduce additional uncertainty into the fitted compositional depth profiles at each fluence.

Non-linear curve fitting was performed using the Levenberg-Marquardt damped least squares fitting algorithm in Origin Pro 9.1.

Interatomic potential for MD simulations. The potentials used for the Ga-Ga, Ga-Sb, and Sb-Sb are of hybrid Abell-Tersoff/ZBL form[4,5]. Here, we summarize the functional form of the potential and provide the values used in our simulations for the various parameters. In LAMMPS, the form of the Tersoff potential is

$$V_{ij}^{Tersoff}(r_{ij}) = f_C(r_{ij})\left[A\exp(-\lambda_1 r_{ij}) - b_{ij}B\exp(-\lambda_2 r_{ij})\right] \quad \text{(S9)}$$

$$f_C(r) = \begin{cases} 1, & r < R - D \\ \frac{1}{2}\left[1 - \sin\left(\frac{\pi(r-R)}{2D}\right)\right], & R - D < r < R + D \\ 0, & r > R + D \end{cases} \quad \text{(S10)}$$

$$b_{ij} = \left(1 + \beta^n \zeta_{ij}^n\right)^{-\frac{1}{2n}} \quad \text{(S11)}$$

$$\zeta_{ij} = \sum_{k \neq i,j} f_C(r_{ik})\, g(\theta_{ijk}) \exp\left[\lambda_3^m (r_{ij} - r_{ik})^m\right] \quad \text{(S12)}$$

$$g(\theta) = \gamma_{ijk}\left\{1 + \frac{c^2}{d^2} - \frac{c^2}{[d^2 + (\cos\theta - \cos\theta_0)^2]}\right\} \quad \text{(S13)}$$

This is connected to the ZBL potential with a Fermi-like switching function:

$$V_{ij}^{net}(r_{ij}) = [1 - f_F(r_{ij})]V_{ij}^{ZBL} + f_F(r_{ij})V_{ij}^{Tersoff} \quad \text{(S14)}$$

$$V_{ij}^{ZBL}(r_{ij}) = \frac{1}{4\pi\epsilon_0}\frac{Z_1 Z_2 e^2}{r_{ij}}\phi_{ZBL}\left(\frac{r_{ij}}{a}\right) \quad \text{(S15)}$$

$$f_F(r) = \frac{1}{1 + \exp[-A_F(r - r_C]} \quad \text{(S16)}$$

The parameters in Supplementary Equations S9 through S16 are given in **Supplementary Table S1**. We indicate the changes we have made to model Sb-Sb interactions, since the original potential of Albe and co-workers was parameterized for As-As interactions. Since As and Sb share the same crystal structures, this is a reasonable approach in the absence of Sb-specific potentials in the literature. The revised parameters for Sb-Sb interactions lead to a predicted α-Sb structure with a cohesive energy of 2.72 eV and a density of 6.71 g/cm^3, in excellent agreement with experiment[6] (6.68 g/cm^3). This is a notable improvement over the potential developed by Norris et al[7], which also modified the parameters of Albe and co-workers but predicted an over-dense α-Sb structure with a density of 7.28 g/cm^3. Since our simulations predict the formation of pure-Sb phases, it is important that these basic physical properties are correctly predicted by the interatomic potential.

Physical properties for additional crystal structures of Sb are given in **Supplementary Table S2**. It is noteworthy that the simple cubic structure has a cohesive energy only slightly smaller in magnitude than that of α-Sb (2.70 eV) but has a slightly higher density (6.85 g/cm^3), which means that it may be favourable to form under high-pressure conditions. This explains the observation of simple cubic crystalline ordering (main text, **Figure 2**) instead of rhombohedral ordering, since phases within the bulk GaSb are compressed by the surrounding material.

Finally, the potential was validated by measuring the simulated sputtering yield for 349 consecutive impacts of 500 eV Kr^+ ions into initially crystalline GaSb(110) on a smaller scale ($\Phi = 3.5 \times 10^{14}$ cm^{-2}). The total sputtering yield was found as 2.3 ± 0.1 atoms/ion, with a split of 1.1 ± 0.1 Ga atoms/ion and 1.2 ± 0.1 Sb atoms/ion. The observation of slight preferential sputtering of Sb is in agreement with experimental results[8], and in contrast to the results of Norris et al, who measured strong preferential sputtering of Ga in their own simulations[7].

Simulated surface construction details with the altered compositional depth profile. The surface with an altered compositional depth profile is constructed in a layer-by-layer manner, where each layer is successively amorphized and thermalized. Since this procedure is not well known in the literature, here we describe it in detail. Each stage of the surface construction procedure is illustrated in **Supplementary Figure S4**.

The "base" layer upon which all other layers are constructed is simply 12 monolayers (1 ML = 0.22 nm) of GaSb(110) which is not modified in any way, as shown in **Supplementary Figure S4(a)**.

For each successive layer, several steps are repeated. First, atoms are created in the active region according to the specified compositional ratio. All atoms in the lower layers created previously are held fixed. Reflecting walls are applied at the top and bottom of the active layer to

confine the atoms in the z-direction, while the lateral boundaries are periodic. Once this setup is complete, the atoms in the active layer are treated as an NVT ensemble, and are assigned velocities according to a Maxwell-Boltzmann distribution corresponding to a high temperature between 2,500 and 3,500 K. This temperature must be chosen carefully, as it must be sufficiently high – well past the melting temperature – to induce significant mobility in the active layer of atoms, but not so high that phase separation occurs due to the artificially-high pressure within the active volume (which may drive Ga and Sb into separate high-density phases which are not characteristic of ion-irradiated GaSb surfaces). In practice, the correct temperature for each layer is found through trial and error. The simulation is run for 50,000 timesteps (~50 ps – times are not exact due to the use of an adaptive timestep size) at this high temperature to allow complete atomic mixing. After this, the temperature of the active layer is immediately reset to 77 K to solidify the surface. The simulation is run for 30,000 timesteps (~30 ps) at this low temperature. Finally, the newly constructed layer is frozen, and the procedure is repeated for the next layer to be added. **Supplementary Figure S4(b-f)** shows each successive layer after construction, and provides the layer thickness, composition, and the optimized high temperature used to construct the layer.

Once all layers of the surface have been constructed, the entire surface is relaxed for some time to allow the interfaces between layers to reach equilibrium. This relaxation proceeds in several stages. For all stages, the bottom 4 ML of the surface are held fixed, while the next-lowest 4 ML are held at the given temperature by a Berendsen thermostat. For the first stage of relaxation, a reflecting wall is placed 0.22 nm above the top layer of the surface, which is necessary to prevent the surface from rapidly decompressing leading to significant loss of atoms. The simulation is run for 100,000 timesteps (~100 ps) under this condition, with the temperature slowly raised from 0 K to 77 K. **Supplementary Figure S4(g)** shows the condition of the surface after this stage. For the second stage of relaxation, the retaining wall remains in place but is repositioned 0.43 nm upwards, allowing the surface to expand slightly. The simulation is again run for 100,000 timesteps (~100 ps) with the temperature held at 77 K. **Supplementary Figure S4(h)** shows the condition of the surface after this stage. For the third stage, the surface is sufficiently stable that the retaining wall is omitted from the simulation. The simulation is run once more for 100,000 timesteps (~100 ps), with the temperature slowly raised from 77 K to 300 K. Finally, for the last stage of relaxation the surface is simply held at 300 K for 500,000 timesteps (~0.5 ns) to ensure ample time to reach interlayer equilibrium.

We carry out this procedure on our private computational server for a 25×25 nm^2 surface, which is duplicated in a 4×4 pattern to provide the final 100×100 nm^2 surface for use on Blue Waters. We show the finalized surface at full size in **Supplementary Figure S5** to allow the reader to accurately visualize the scale of the simulation. The large dimensions were chosen to be larger than the typical feature size of ion-induced GaSb quantum dots (30 to 80 nm spacing), to capture potential long-scale periodic variations during ion bombardment.

Clustering analysis. Cluster identification and visualization is carried out with OVITO[9]. A cluster is defined as a set of atoms of a single element or type which either (a) do not have nearest neighbours of any other element or type, or (b) have a nearest neighbour which satisfies criterion (a). Here we describe the modifier pipeline used to identify clusters.

First, all atoms of the ion type are deleted ("Select particle type" and "Delete selected particles" modifiers). Next, all particles of the opposite type of the desired cluster element are selected (i.e. to identify Sb clusters, we first select all Ga atoms). The selection is expanded within a cut-off distance ("Expand selection" modifier), which is determined from the potential cut-off for the Ga-Sb interaction and is equal to 3.6 Å. The selection is then inverted ("Invert selection" modifier), and at this point consists of all atoms of the desired element which do not have a nearest neighbour of the opposite type (criterion (a) is satisfied). The selection is then expanded again within the same cut-off distance as before, to include all atoms of the desired element which satisfy criterion (b).

To visualize clusters of only a single atom type, the selection is inverted once more (selecting all particles which are not members of a cluster) and the selected atoms are then deleted. To visualize clusters of multiple atoms types in the same frame (i.e. to see Ga clusters and Sb cluster simultaneously), the "Compute property" modifier is used to assign a unique value to the selected atoms in clusters, and the above procedure is repeated to identify clusters of the other atom type and to assign the same unique property value to those atoms as well. Once this is done, all atoms with the unique property value are selected ("Expression select" modifier), the selection is inverted to select all atoms which are not members of any cluster, and these atoms are deleted. By this means we visualize clusters of both elemental types, as seen in **Figure 3(b)** in the main text.

Cluster formation for other compositional depth profiles. We show snapshots of small (6 nm width) GaSb surfaces with various other compositional depth profiles in **Supplementary Figure S6**. It can readily be seen from these snapshots that phase separation and cluster formation occur for any layer with altered composition, independent of the other layers. This indicates that the clustering seen in **Figure 2** is not a simulation artefact. We also note that other authors have observed similar clustering phenomena from MD simulations of III-V amorphization and melting[4], further supporting this assertion.

Amorphization Analysis. The radial distribution function (RDF) $g_{\Phi}(r)$ at fluence Φ is used to measure the degree of surface amorphization during the simulation. The RDF is obtained from OVITO through its "Coordination analysis" modifier. Since this analysis implies a normalization with respect to the number density of atoms (e.g. number of atoms divided by total simulation cell volume), we renormalize the RDF to the number of atoms in the simulation cell. This allows consistent comparison across fluence steps with varying numbers of atoms. The fixed layer of crystalline GaSb(110) is omitted before obtaining the RDF.

The total difference in RDF of the final and initial structures and the difference between the RDF at fluence Φ and that of the initial structure are defined by Supplementary Equations S17 and S18, respectively.

$$\Delta_{Total} = \sum_{i=RDF\ bins} |g_{final}(r_i) - g_{initial}(r_i)| \quad (S17)$$

$$\Delta_{\Phi} = \sum_{i=RDF\ bins} |g_{\Phi}(r_i) - g_{initial}(r_i)| \quad (S18)$$

From this, we define the percent amorphization at fluence Φ to be the ratio of the two (Supplementary Equation S19).

$$\%Amorphization = \frac{\Delta_{\Phi}}{\Delta_{Total}} \quad (S19)$$

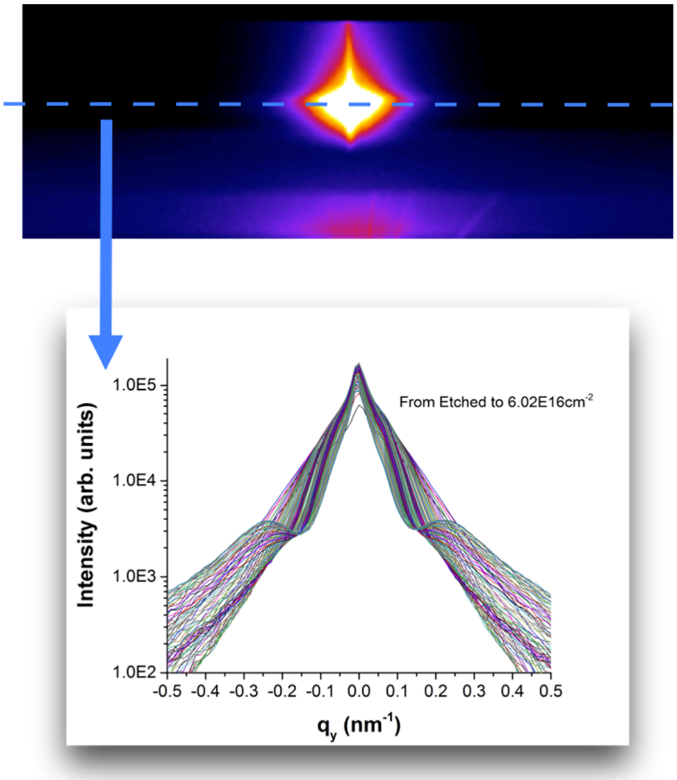

Supplementary Figure S1. Illustration of the 1-D Yoneda wing analysis. The top image shows an example of raw 2-D GISAXS data in the (q_y, q_z) plane. The dotted line indicates the 1-D Yoneda wing. The bottom plot shows the 1-D Yoneda wing values versus q_y for each fluence step.

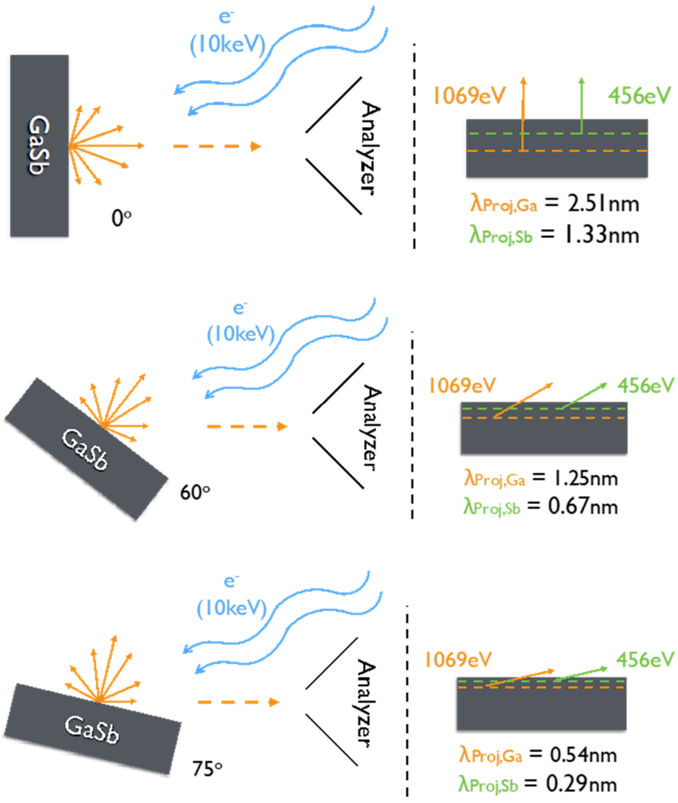

Supplementary Figure S2. Experimental setup for the angle-resolved Auger electron spectroscopy (ARAES) measurements. At right, the mean free paths for Ga and Sb Auger electrons are given, indicating that changing the angular orientation of the sample provides depth-sensitive compositional measurements.

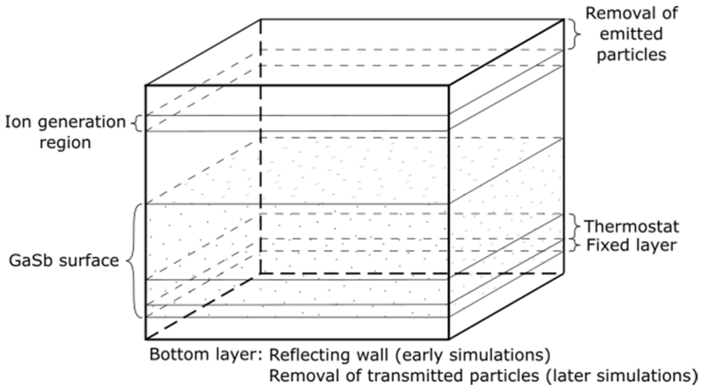

Supplementary Figure S3. Design schematic for the MD simulations. The bottom reflecting wall is used in the simulations of altered-composition GaSb, while the bottom particle removal layer is used in the high-fluence simulations of initially pristine GaSb(110). Note that the thickness of each layer in the diagram is not drawn to scale.

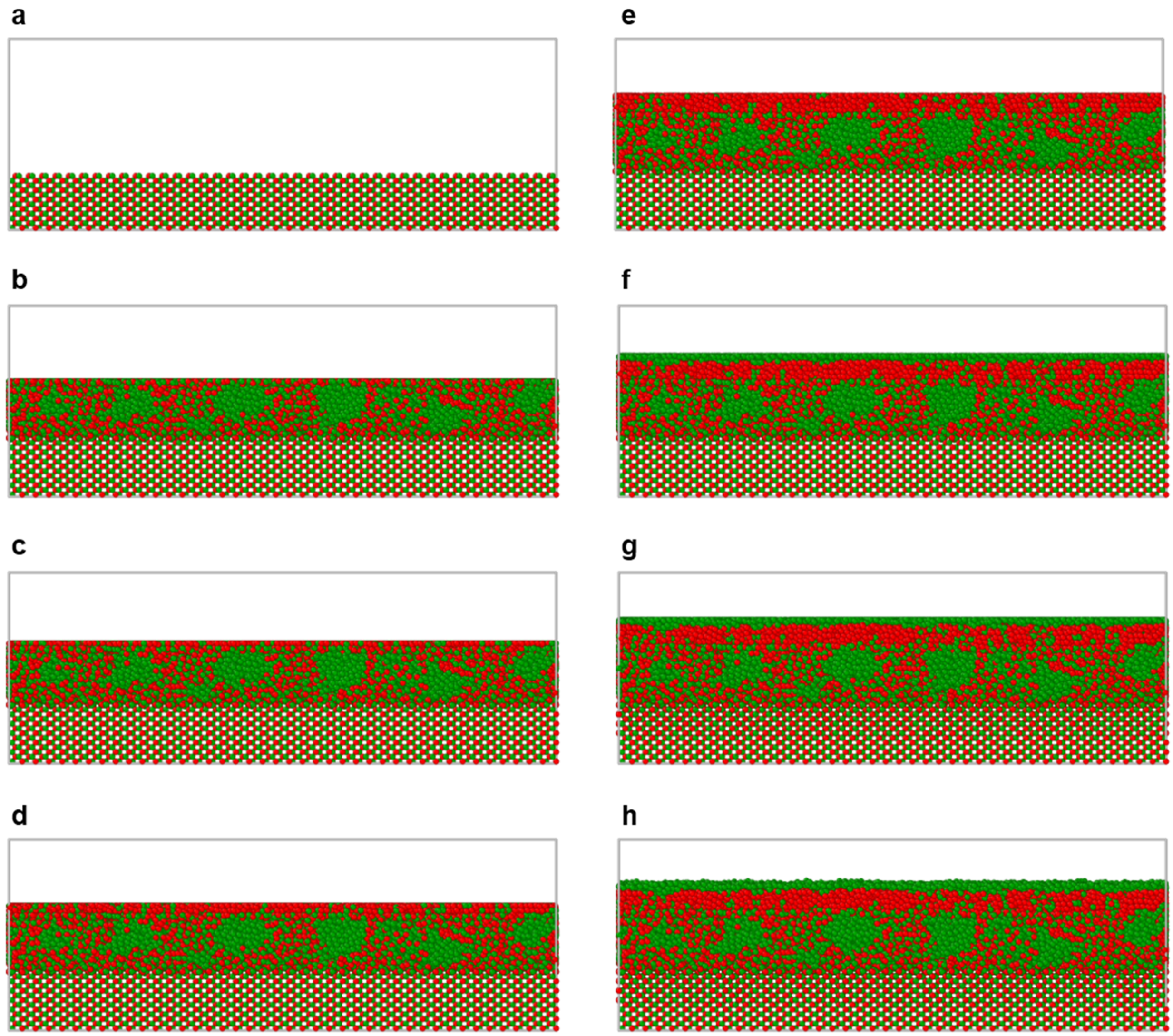

Supplementary Figure S4. Snapshots of the altered-composition GaSb surface after each stage of construction. All snapshots are 25 nm in width. (a) Initial crystalline GaSb(110) layer of 12 ML (2.59 nm) thickness. (b) Added layer with 62.5% Sb composition and 12 ML (2.59 nm) thickness, heated at 2,500 K. (c) Added layer with 65% Ga composition and 1 ML (0.22 nm) thickness, heated at 3,000 K. (d) Added layer with 80% Ga composition and 1 ML (0.22 nm) thickness, heated at 2,500 K. (e) Added layer with 90% Ga composition and 2 ML (0.43 nm) thickness, heated at 2,000 K. (f) Added layer with 100% Sb composition and 1 ML (0.22 nm) thickness, heated at 2,500 K. (g) Entire surface after first stage of relaxation with slow heating from 0 to 77 K. (h) Entire surface after second stage of relaxation while held at 77 K. Snapshots were created with the OVITO software tool[9].

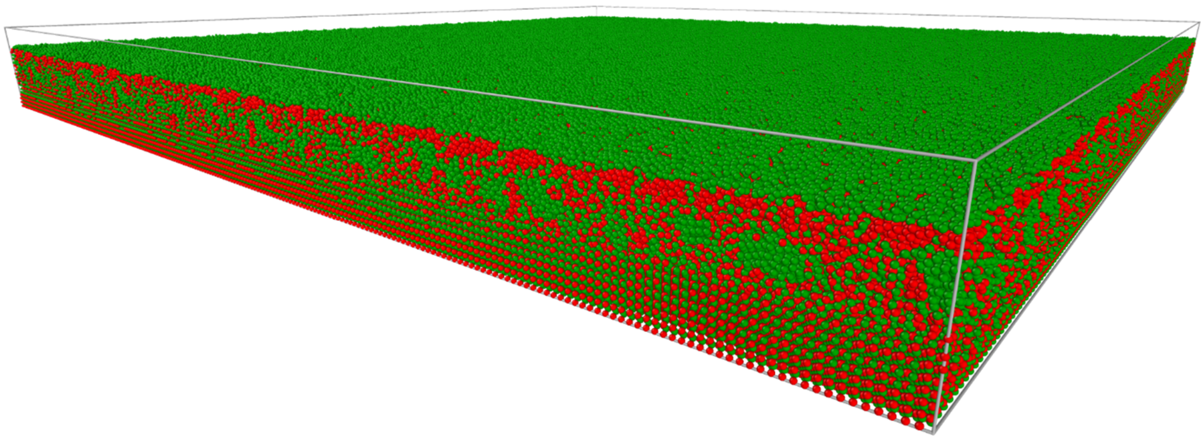

Supplementary Figure S5. Snapshot of the final altered-composition GaSb surface. The total simulation cell volume is 100 × 100 × 8.71 nm^3. The pictured surface is constructed from a 4 × 4 duplication of the 25 × 25 nm^2 surface (**Supplementary Figure S4**) after all relaxation stages are completed. Snapshot was created with the OVITO software tool[9].

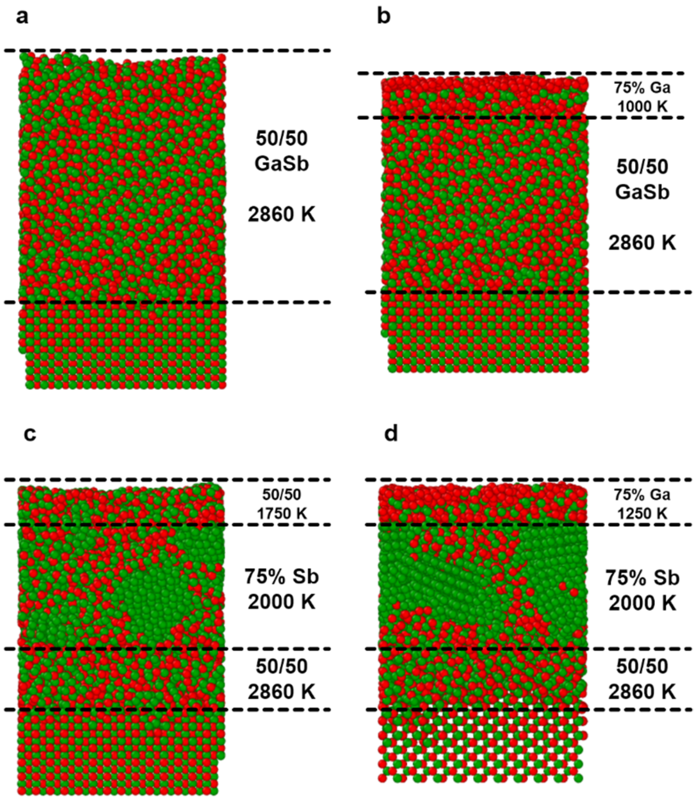

Supplementary Figure S6. Snapshots of small (6 nm width) GaSb surfaces constructed with varied compositional depth profiles. Each surface is built with the same layer-by-layer procedure as the surface shown in **Supplementary Figure S5**. Layer compositions and heating temperatures are given in the figure. (a) Simple 50/50 amorphous GaSb surface. (b) Surface with a 75% Ga top layer. (c) Surface with a 75% Sb subsurface layer. (d) Surface with both 75% Ga and 75% Sb layers. Snapshots were created with the OVITO software tool[9].

Supplementary Table S1: Parameter sets for the Tersoff/ZBL hybrid potential used to model GaSb in LAMMPS. Parameters for the Sb-Sb interaction in bold italic font are changed from their original values[4] for As-As interactions.

**Parameter**	**Ga-Ga**	**Ga-Sb**	**Sb-Sb**
Two-body parameters			
A (eV)	535.199	2521.76	***22752.7***
B (eV)	410.132	544.904	***412.687***
λ_1 (Å^{-1})	1.60916	2.50245	***3.31331***
λ_2 (Å^{-1})	1.44970	1.74517	***1.56288***
Three-body parameters			
m	1	3	1
γ	0.007874	1.0	0.455
λ_3 (Å^{-1})	1.846	0.968688	3.161
c	1.918	1.20875	0.1186
d	0.75	0.839761	0.1612
$\cos(\theta_0)$	-0.3013	-0.427706	-0.07748
n	1.0	4.60221	1.0
β	1.0	0.363018	1.0
Cut-off parameters			
R (Å)	2.95	3.5	3.6
D (Å)	0.15	0.1	0.2
ZBL parameters			
Z_1	31	31	51
Z_2	31	51	51
r_C (Å)	1.2	1.2	1.2
A_F (Å^{-1})	12.01	12.01	12.01

Supplementary Table S2: Calculated properties for several crystal structures of Sb calculated with the modified Tersoff potential described in the main text. Note that listing a crystal structure here does not necessarily imply that it is expected to be observed in experiments.

**Lattice structure**	**Lattice constants (Å)**	**Cohesive energy (eV)**	**Density (g cm^{-3})**
Rhombohedral (α-Sb)	$a = 4.32, c = 11.19$	2.72	6.71
Simple cubic (sc-Sb)	$a = 3.09$	2.70	6.85
Face-centred cubic (fcc-Sb)	$a = 4.69$	2.57	7.84
Body-centred cubic (bcc-Sb)	$a = 3.70$	2.44	7.99
Diamond cubic (dc-Sb)	$a = 6.98$	2.27	4.75

Supplementary References

1. Zhou, H., Zhou, L., Özaydin, G., Ludwig, K. F. & Headrick, R. L. Mechanisms of pattern formation and smoothing induced by ion-beam erosion. *Phys. Rev. B - Condens. Matter Mater. Phys.* **78**, 165404 (2008).
2. Sigmund, P. & Lam, N. Alloy and isotope sputtering. *K. Dan. Vidensk. Selsk. Det Mat.-Fys. Meddelelser* **43**, 255–349 (1993).
3. A. Savitsky & Golay, M. J. Smoothing and differentiation of data by simplified least squares procedures. *Anal. Chem.* **36**, 1627–1639 (1964).
4. Albe, K., Nordlund, K., Nord, J. & Kuronen, A. Modeling of compound semiconductors: Analytical bond-order potential for Ga, As, and GaAs. *Phys. Rev. B - Condens. Matter Mater. Phys.* **66**, 035205 (2002).
5. Powell, D., Migliorato, M. A. & Cullis, A. G. Optimized Tersoff potential parameters for tetrahedrally bonded III-V semiconductors. *Phys. Rev. B - Condens. Matter Mater. Phys.* **75**, 115202 (2007).
6. *CRC Handbook of Chemistry and Physics*. (CRC Press/Taylor and Francis, 2017).
7. Norris, S. A., Samela, J., Vestberg, M., Nordlund, K. & Aziz, M. J. Crater functions for compound materials: A route to parameter estimation in coupled-PDE models of ion bombardment. *Nucl. Instrum. Methods Phys. Res. Sect. B Beam Interact. Mater. At.* **318**, 245–252 (2014).
8. El-Atwani, O., Gonderman, S. & Paul Allain, J. Near sputter-threshold GaSb nanopatterning. *J. Appl. Phys.* **114**, (2013).
9. Stukowski, A. Visualization and analysis of atomistic simulation data with OVITO–the Open Visualization Tool. *Model. Simul. Mater. Sci. Eng.* **18**, 015012 (2010).
